# Editorial: Anaerobic digestion of waste organics: toxicity and management

**DOI:** 10.3389/fmicb.2023.1243205

**Published:** 2023-07-18

**Authors:** Fang Zhang, Huijie Hou, Jie Fu, Juan-Rodrigo Bastidas-Oyanedel

**Affiliations:** ^1^College of Resources and Environment, Fujian Agriculture and Forestry University, Fuzhou, China; ^2^School of Environmental Science and Engineering, Huazhong University of Science and Technology, Wuhan, China; ^3^SDU-Department of Chemical Engineering, Biotechnology, and Environmental Technology, University of Southern Denmark, Odense, Denmark

**Keywords:** anaerobic digestion, waste organics, methane, methanogens, toxicity

As an important anaerobic environmental biotechnology, anaerobic digestion (AD) can convert biodegradable organic waste, including organic wastewaters, waste-activated sludge, and food waste, into biogas (Dai et al., 2020; Toutian et al., 2020). AD involves cascading steps of hydrolysis/acidogenesis, acetogenesis/homoacetogensis, and methanogenesis, in which the distinct different microorganisms involved include acidogenic bacteria, acetogens, homoacetogens, and hydrogenotrophic and aceticlastic methanogens (Saha et al., 2020). This method offers several advantages, including low running costs, adaptability to various feedstock or conditions, and the lack of need for sterilization. However, toxicants present in organic wastes, such as antibiotics, metal ions, nanoparticles, and microplastic fibers, may hinder the overall performance of AD (Mu and Chen, 2011; Hart et al., 2022; Tang et al., 2023).

The present Research Topic “*Anaerobic digestion of waste organics: toxicity and management*” aims to cover promising and novel research into biological strategies for improving AD performance, especially physicochemical analyses and multi-omics technologies. This topic comprises four original articles contributed by 23 authors. Propionic acid (HPr) is known as a frequently accumulated intermediate in anaerobic digesters due to the thermodynamical limitation. Thus, Kim et al. identify key players as metagenome-assembled genomes in HPr oxidation and organic overloading recovery in anaerobic digesters. The results showed that at least two key species of *JABUEY01 sp013314815* and *Methanoculleus sp002497965* are responsible for efficient propionate removal, which can be used as microbial cocktails for the stable operation of AD. The conductive materials of biochar and activated carbon were capable of improving methane production from organic waste via direct interspecific electron transfer (DIET) between acidogens and methanogens. Thus, Abid et al. investigated the impact of biochar on the anaerobic degradation of olive mill wastewater. The results demonstrated that adding biochar to olive mill wastewater increased the methane yield by 97.8%. The microbial diversity revealed that biochar supplementation significantly increased the abundance of the genera *Methanothrix* and *Methanosarcina* potentially involved in DIET. Meanwhile, Wu et al. studied the effects of activated carbon- and graphite-conductive media on methane production in WAS fermentation. Their results show that the largest biogas yield in a 100 mesh-activated carbon group was 468.2 ml/g VSS, which was 13.8% higher than the blank group. The three genera *Nitrososphaeraceae, Methanobacterium*, and *Methanosaeta* were cultured in the activated carbon and graphite groups. On the other hand, mitigating CH_4_ emissions may reduce the current global warming effects due to its high global warming potential. Thus, Im et al. reported that adding salt to the pig slurry storage tank can inhibit the activity of methanogens and reduce unwanted CH_4_ emissions. More CH_4_ can be obtained in the following biogas production from the stored pig slurry due to the preservation of organics.

In summary, the articles included in this Research Topic demonstrate the importance of toxicity and management in the anaerobic digestion of waste organics. Additionally, physicochemical analyses potentially augmented by omics technologies are shown to be useful tools for providing solid evidence and revealing the potential mechanism. The outcome of such studies may improve understanding of the toxicity and management of organic waste degradation and subsequently help improve the development of anaerobic fermentation.

## Author contributions

FZ, HH, JF, and J-RB-O wrote the manuscript. All authors read and approved the final manuscript.
